# CXCR2 Signaling Protects Oligodendrocytes and Restricts Demyelination in a Mouse Model of Viral-Induced Demyelination

**DOI:** 10.1371/journal.pone.0011340

**Published:** 2010-06-28

**Authors:** Martin P. Hosking, Emanuele Tirotta, Richard M. Ransohoff, Thomas E. Lane

**Affiliations:** 1 Department of Molecular Biology and Biochemistry, University of California Irvine, Irvine, California, United States of America; 2 Institute for Immunology, University of California Irvine, Irvine, California, United States of America; 3 Sue and Bill Gross Stem Cell Center, University of California Irvine, Irvine, California, United States of America; 4 Neuroinflammation Research Center, Department of Neuroscience, Lerner Research Institute, Cleveland Clinic, Cleveland, Ohio, United States of America; Boston University School of Medicine, United States of America

## Abstract

**Background:**

The functional role of ELR-positive CXC chemokines during viral – induced demyelination was assessed. Inoculation of the neuroattenuated JHM strain of mouse hepatitis virus (JHMV) into the CNS of susceptible mice results in an acute encephalomyelitis that evolves into a chronic demyelinating disease, modeling white matter pathology observed in the human demyelinating disease Multiple Sclerosis.

**Methodology/Principal Findings:**

JHMV infection induced the rapid and sustained expression of transcripts specific for the ELR (+) chemokine ligands CXCL1 and CXCL2, as well as their binding receptor CXCR2, which was enriched within the spinal cord during chronic infection. Inhibiting CXCR2 signaling with neutralizing antiserum significantly (p<0.03) delayed clinical recovery. Moreover, CXCR2 neutralization was associated with an increase in the severity of demyelination that was independent of viral recrudescence or modulation of neuroinflammation. Rather, blocking CXCR2 was associated with increased numbers of apoptotic cells primarily within white matter tracts, suggesting that oligodendrocytes were affected. JHMV infection of enriched oligodendrocyte progenitor cell (OPC) cultures revealed that apoptosis was associated with elevated expression of cleaved caspase 3 and muted Bcl-2 expression. Inclusion of CXCL1 within JHMV infected cultures restricted caspase 3 cleavage and increased Bcl-2 expression that was associated with a significant (p<0.001) decrease in apoptosis. CXCR2 deficient oligodendrocytes were refractory to CXCL1 mediated protection from JHMV – induced apoptosis, readily activating caspase 3 and down regulating Bcl-2.

**Conclusion/Significance:**

These findings highlight a previously unappreciated role for CXCR2 signaling in protecting oligodendrocyte lineage cells from apoptosis during inflammatory demyelination initiated by viral infection of the CNS.

## Introduction

How chemokine receptor signaling contributes to chronic neurologic diseases has largely been considered within the context of targeted leukocyte recruitment into the CNS [1]. However, numerous resident cell types of the CNS express chemokine receptors under non-inflammatory and inflammatory conditions (reviewed in [2], [3]), indicating that these cells are capable of responding to specific chemokine ligands. Thus, chemokine signaling may participate in either repair and/or exacerbation of pathology following insult, injury, or infection of the CNS [4]–[6]. CXCR2, the receptor for the murine ELR+ CXC chemokine ligands CXCL1,−2,−3, −5, −6 and −7, has been detected either *in vitro* or *in vivo* upon resident cells of the CNS, including oligodendrocyte precursor cells (OPC) [7]–[12]. Expression of CXCR2 on OPC is critical in the positional migration of these cells during development and axon myelination [12]–[14], and CXCR2 has been suggested to be important in regulating remyelination following experimental demyelination [5], [6], [15]. Additionally, in active MS plaque lesions CXCR2 has been found upon proliferating oligodendrocytes and reactive astrocytes, while its ligand CXCL1 has been associated with activated astrocytes [10], [11].

Following experimental infection with the neurotropic viruses including the JHM strain of mouse hepatitis virus (JHMV) or Theiler's murine encephalomyelitis virus (TMEV), both CXCL1 and CXCL2 are up regulated within the CNS. Moreover, *in vitro* experiments revealed that JHMV - and TMEV - infected astrocytes readily express CXCL1 and CXCL2, suggesting that astrocytes are the primary source for these chemokines *in vivo* [16], [17]. The presence of CXCR2 and/or signaling ligands during numerous CNS inflammatory pathologies implies a potential role for CXCR2 signaling in protection or disease progression, yet a functional role for ELR+ chemokines and/or CXCR2 in a chronic virus - induced neuroinflammatory disease remains incompletely understood. In particular, the relative activities of the ELR+ chemokines towards leukocytes and resident neuroepithelial cells have not been differentiated.

The present study was undertaken to characterize, for the first time, the functional role of ELR+ chemokines during chronic viral infection of the CNS. Inoculation with JHMV into the CNS of susceptible strains of mice results in an acute encephalomyelitis characterized by wide spread infection of astrocytes, microglia, and oligodendrocytes and relative sparing of neurons [18]. Although a robust cell-mediated immune response occurs during acute disease [19], [20], sterilizing immunity is not achieved, and JHMV persists primarily within oligodendrocytes [21]. Histological features associated with viral persistence include the development of an immune-mediated demyelinating disease analogous to the human demyelinating disease multiple sclerosis (MS), with both T cells and macrophages important in amplifying disease severity by contributing to myelin damage [22], [23]. Using JHMV infection as a model of immune-mediated demyelination, we demonstrate a previously unappreciated role for ELR-positive chemokines in protecting cells of the oligodendrocyte lineage from apoptosis during viral – induced demyelination.

## Results

### CXCR2 and its ligands CXCL1 and CXCL2 are upregulated during viral – induced demyelination

**Figure 1:**
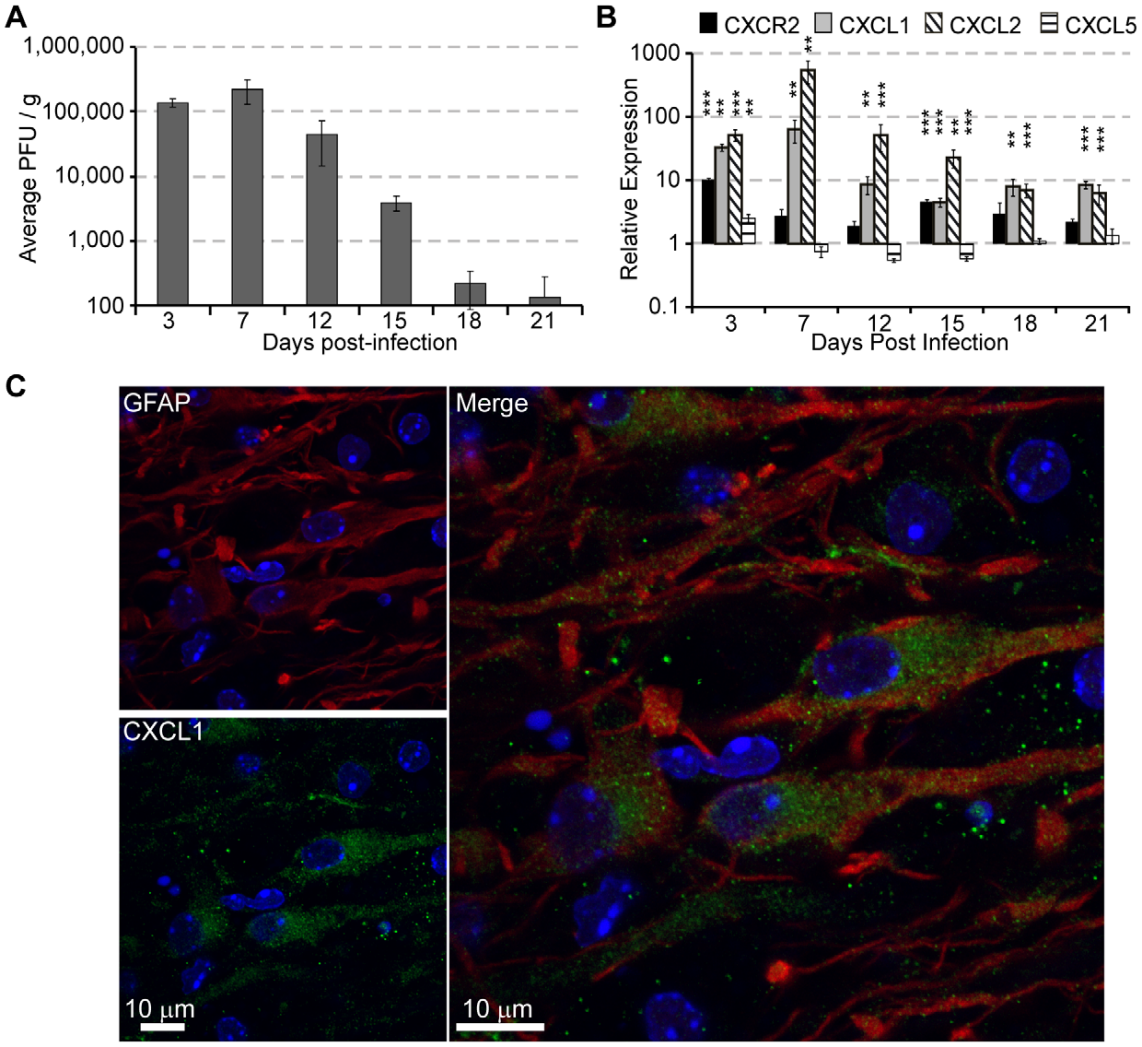
CXCR2 and ELR+ chemokines are upregulated within the spinal cord during chronic JHMV infection. C57BL/6 mice were i.c. infected with 500 pfu JHMV and spinal cords removed at defined times p.i. to assess viral burden (**A**) and transcripts specific for CXCR2, CXCL1, CXCL2, and CXCL5 using semi-quantitative real time PCR (**B**). For PCR results, data represents the fold induction relative to sham infected mice (n = 3–5, *p<0.05, **p<0.01, ***p<0.001). Representative immunofluorescence of the spinal cord white matter at 15 days p.i reveals CXCL1 expression within reactive hypertrophic GFAP+ astrocytes (**C**).

To investigate the role of CXCR2 signaling during chronic – viral encephalomyelitis, C57BL/6 mice were intracerebrally (i.c.) inoculated with JHMV, and the mRNA expression pattern of CXCR2, as well as its associated ELR+ chemokines CXCL1, CXCL2, and CXCL5, was assessed within the spinal cord, the primary site of demyelination during chronic disease. Within the spinal cord, JHMV viral loads peaked at 1 week post-infection (p.i) before declining to below the level of detection (100 pfu/g) by day 18 p.i. (**Figure 1A**). Significantly (*p*≤0.01) elevated mRNA transcripts for both CXCL1 and CXCL2 were observed throughout infection compared to sham-infected control mice, and expression was independent of viral load (**Figure 1B**). Immunostaining for CXCL1 revealed that GFAP+ astrocytes are the primary cellular source of CXCL1 within the spinal cord (**Figure 1C**). The prolonged and elevated expression of the CXCR2 ligands CXCL1 and CXCL2 mRNA throughout the course of JHMV infection within the spinal cord, coupled with the localized CXCL1 expression within spinal cord white matter, implicate a potential role for CXCR2 signaling in host defense during chronic JHMV infection.

### Blocking CXCR2 increases the severity of clinical disease

**Figure 2:**
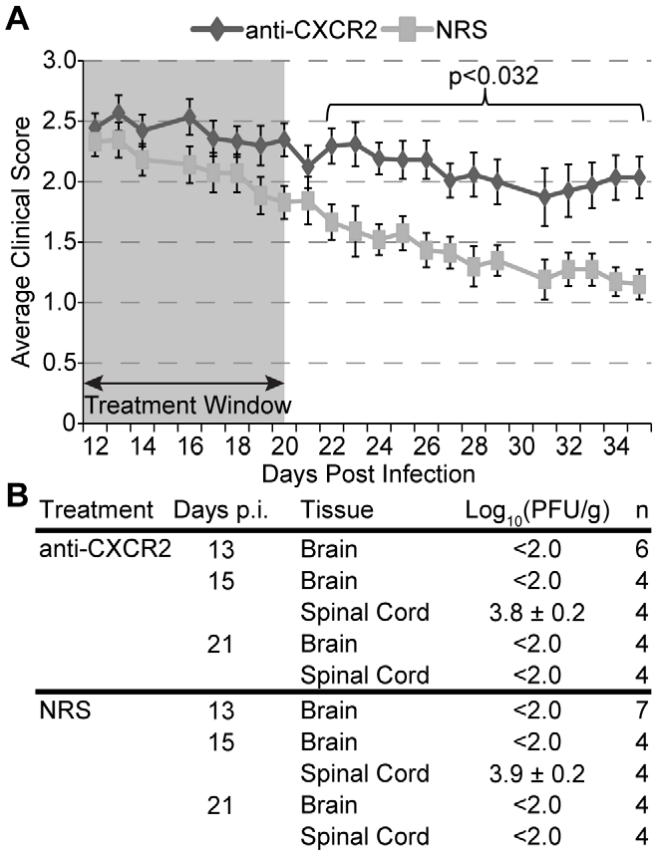
CXCR2 neutralization mutes spontaneous recovery from JHMV induced demyelination. C57BL/6 mice were i.c. infected with 500 pfu JHMV and treated with neutralizing CXCR2 antiserum or control normal rabbit serum (NRS) i.p. from day 12 to 20, indicated by the shaded box. Treated mice were allowed to recover for a further two weeks and clinical severity was assessed (**A**). In addition, brains and spinal cords from treated mice were removed at the beginning, middle, and end of treatment to assess JHMV viral burden (**B**). For panel ***A*** data is a summation of four independent experiments (anti-CXCR2 n = 32; NRS n = 31). For panel ***B*** data is a summation of two independent experiments.

**Figure 3:**
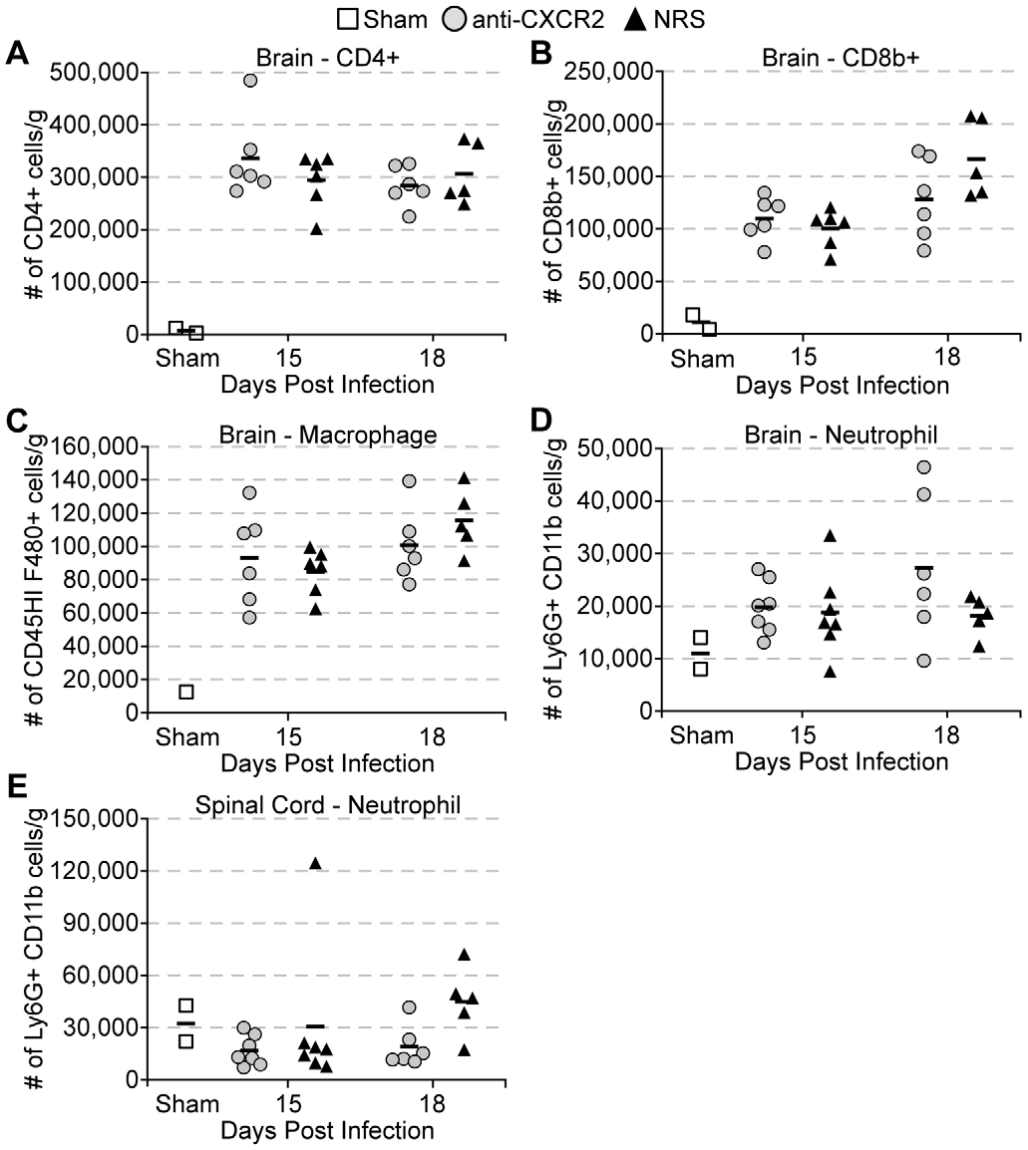
CXCR2 neutralization does not alter neuroinflammation during chronic JHMV infection. C57BL/6 mice were i.c. infected with 500 pfu JHMV and treated with neutralizing CXCR2 antiserum or control normal rabbit serum (NRS) i.p. from day 12 to 20. Brains (**A–D**) and/or spinal cords (**E**) were removed from perfused mice at defined times p.i. and were assessed for CD4+ (**A**), CD8+ T lymphocyte (**B**), macrophage (**C**), and neutrophil (**D & E**) accumulation within the CNS. Data in panels ***A***–***C*** are representative of four independent experiments, and data in panels *D & E* are representative of two independent experiments.

**Figure 4:**
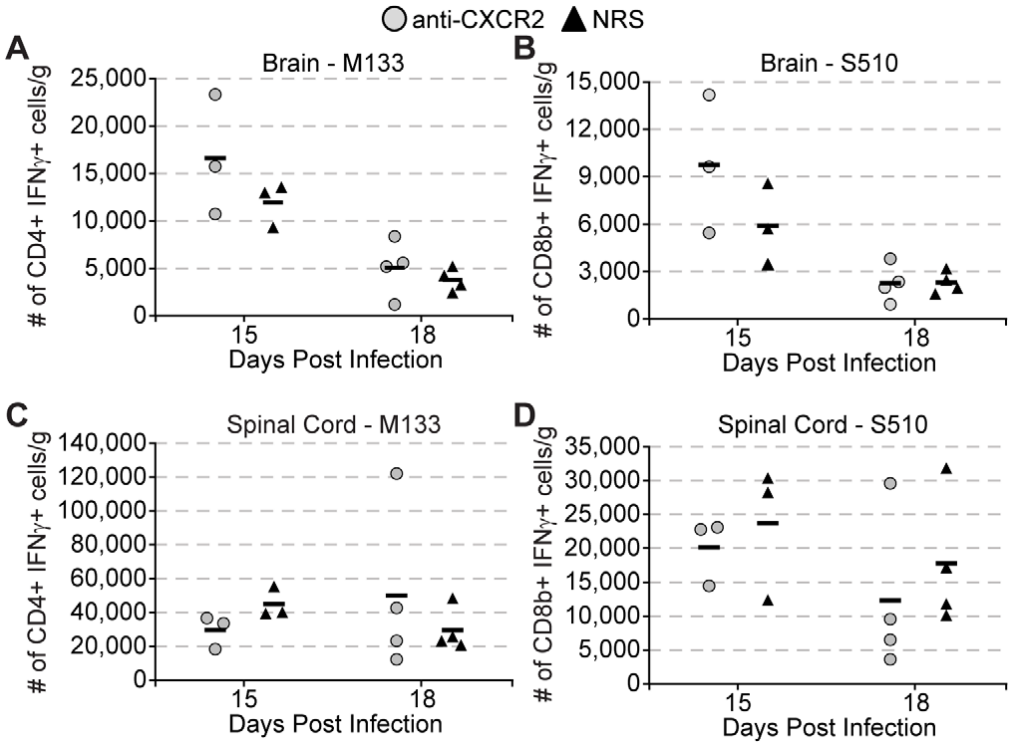
CXCR2 neutralization does not diminish virus – specific T cell infiltration during chronic JHMV infection. C57BL/6 mice were i.c. infected with 500 pfu JHMV and treated with neutralizing CXCR2 antiserum or control normal rabbit serum (NRS) i.p. from day 12 to 18. Brains (**A** & **B**) and spinal cords (**C** & **D**) were removed at defined times p.i. and isolated cells were stimulated *ex vivo* for six hours with 5 µM of the immunodominant CD4+ epitope M_133–147_ (**A** & **C**) or 5 µM of the immunodominant CD8+ epitope S_510–518_ (**B** & **D**) before intracellular IFNγ staining was assessed. Data is representative of two independent experiments.

To assess a functional role for signaling by ELR+ chemokines during chronic viral – induced demyelination, JHMV infected C57BL/6 mice were treated with CXCR2 neutralizing antiserum or control serum (NRS) from days 12–20 p.i, after which mice were allowed to recover until day 35 p.i. Administration of anti-CXCR2 treatment resulted in sustained clinical disease compared to mice treated with control antiserum (**Figure 2A**). CXCR2 neutralization did not influence JHMV viral loads within either the brain or spinal cord during chronic infection, as determined by measuring viral titers by plaque assay at defined times post-treatment (**Figure 2B**). In addition, immunophenotyping the cellular infiltrate by flow cytometry in mice treated with anti-CXCR2 revealed that the frequency and total numbers of CD4+ T lymphocytes, CD8+ T lymphocytes, infiltrating macrophages (CD45^high^/F480+), and neutrophils (Ly6G+ CD11b+) were similar when compared to mice treated with control serum (**Figure 3** **and Table S1**). Correspondingly, chemokine mRNA expression within the CNS was not affected (**Figure S1**). Moreover, blocking CXCR2 did not affect total numbers of virus-specific CD4+ or CD8+ T cells, as determined by intracellular IFN-γ staining to defined immunodominant peptides, when compared to mice treated with control serum (**Figure 4**). Therefore, the increase in clinical severity in mice treated with anti-CXCR2 was neither the result of viral recrudescence, nor alteration in the composition or frequency of infiltrating immune cells.

### Blocking CXCR2 increases the severity of demyelination associated apoptosis in white matter tracts

**Figure 5:**
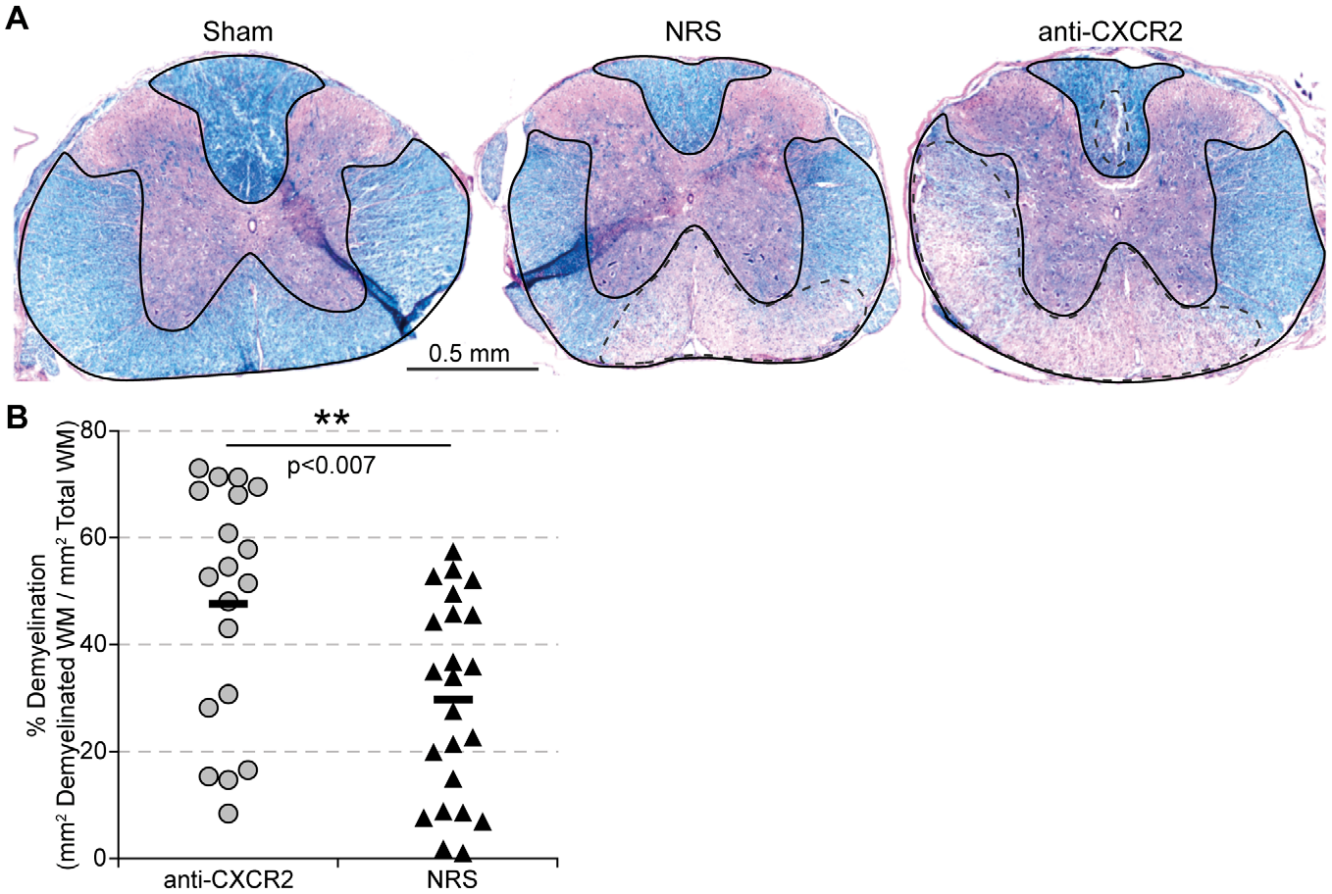
CXCR2 neutralization increases white matter demyelination. C57BL/6 mice were i.c. infected with 500 pfu JHMV and treated with neutralizing CXCR2 antiserum or control normal rabbit serum (NRS) i.p. from day 12 to 20. Spinal cords were removed at day 35 p.i. and coronal sections along the length of the spinal cord were stained with luxol fast blue to assess myelin integrity (**A**). Quantification of the total area of demyelination along the entire length of the spinal cord (**B**) revealed a significant increase in the percent demyelinated area of mice treated with CXCR2 antiserum (solid outline indicates total white matter, dashed outline indicates area of demyelinated white matter). Data is a summation of four independent experiments (anti-CXCR2 n = 19, NRS n = 23).

**Figure 6:**
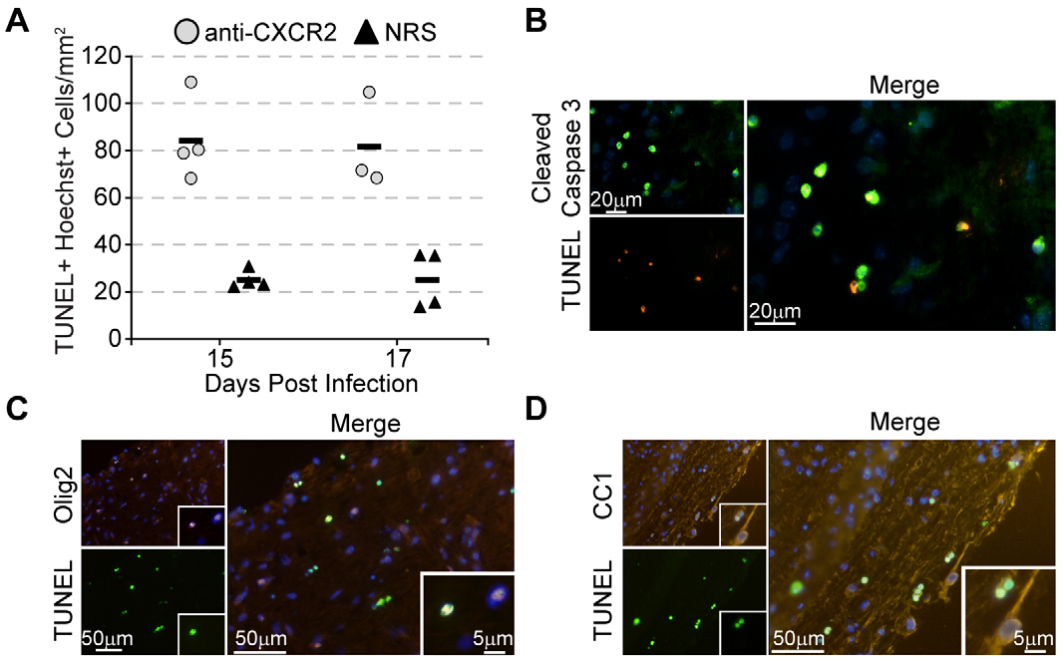
CXCR2 neutralization increases oligodendrocyte lineage cell apoptosis in the spinal cord. C57BL/6 mice were i.c. infected with 500 PFU JMHV and were treated with CXCR2 neutralizing antiserum or control normal rabbit serum (NRS) every other day beginning at day 12 p.i. Mice were sacrificed at day 15 and 17 p.i. and spinal cords were removed and processed for TUNEL labeling. (**A**) Quantification of the number of white matter TUNEL-positive nuclei revealed an approximate 3 to 4 fold increase in the number of apoptotic cells in anti-CXCR2 treated mice (*** *p*<0.001) compared to NRS-treated mice at days 15 and 17 p.i. (**B**) Co-immuno fluorescence of TUNEL with cleaved caspase 3 confirmed that TUNEL reactive cells were indeed apoptotic. Immunophenotyping the TUNEL-positive cells revealed apoptotic (**C**) CC1^+^ oligodendrocytes and (**D**) Olig2^+^ oligodendrocyte precursor cells. Data presented is representative of two independent experiments. Bars: (**B**) 20 µm; (**C & D**) 50 µm; (**C & D**) inset 5 µm.

Quantification of demyelination at day 35 p.i. across the length of the spinal cord revealed a significant (*p*≤0.007) increase in the severity of demyelination within mice receiving CXCR2 antiserum compared to those receiving control serum (**Figure 5A and B**). One potential explanation for the worsened demyelination would be that oligodendrocytes were undergoing programmed cell death. Therefore we next determined whether mice treated with anti-CXCR2 displayed an increase in apoptotic cells within the CNS compared to control-treated mice. CXCR2 neutralization significantly (*p*≤0.001) enhanced the number of TUNEL-reactive nuclei at days 15 and 17 p.i. within the white matter parenchyma of treated mice compared mice treated with control serum (**Figure 6A**). Regions of TUNEL-positive cells were restricted to white matter tracts, and both the area and density of TUNEL reactivity was increased within anti-CXCR2 treated mice. Greater than 90% of the TUNEL reactive cells also co-localized with cleaved caspase 3, indicating that the cells within the white matter of mice receiving CXCR2 antiserum were undergoing an apoptotic and not necrotic death (**Figure 6B**). Immunofluorescence staining for the oligodendrocyte precursor marker Olig 2 (**Figure 6C**) and the oligodendrocyte marker CC1 (**Figure 6D**), revealed apoptotic oligodendrocyte lineage cells within the white matter of anti-CXCR2 treated mice. The increase in apoptosis did not appear to be a direct causal result of viral persistence within an individual cell, as similar frequencies of apoptotic cells containing JHMV nucleocapsid antigen were observed in both experimental groups of mice (**data not shown**). These data collectively indicate that CXCR2 signaling during chronic JHMV infection enhances protection of cells of the oligodendrocyte lineage from apoptosis.

### CXCR2 signaling reduces JHMV – induced oligodendrocyte apoptosis *in vitro*

**Figure 7:**
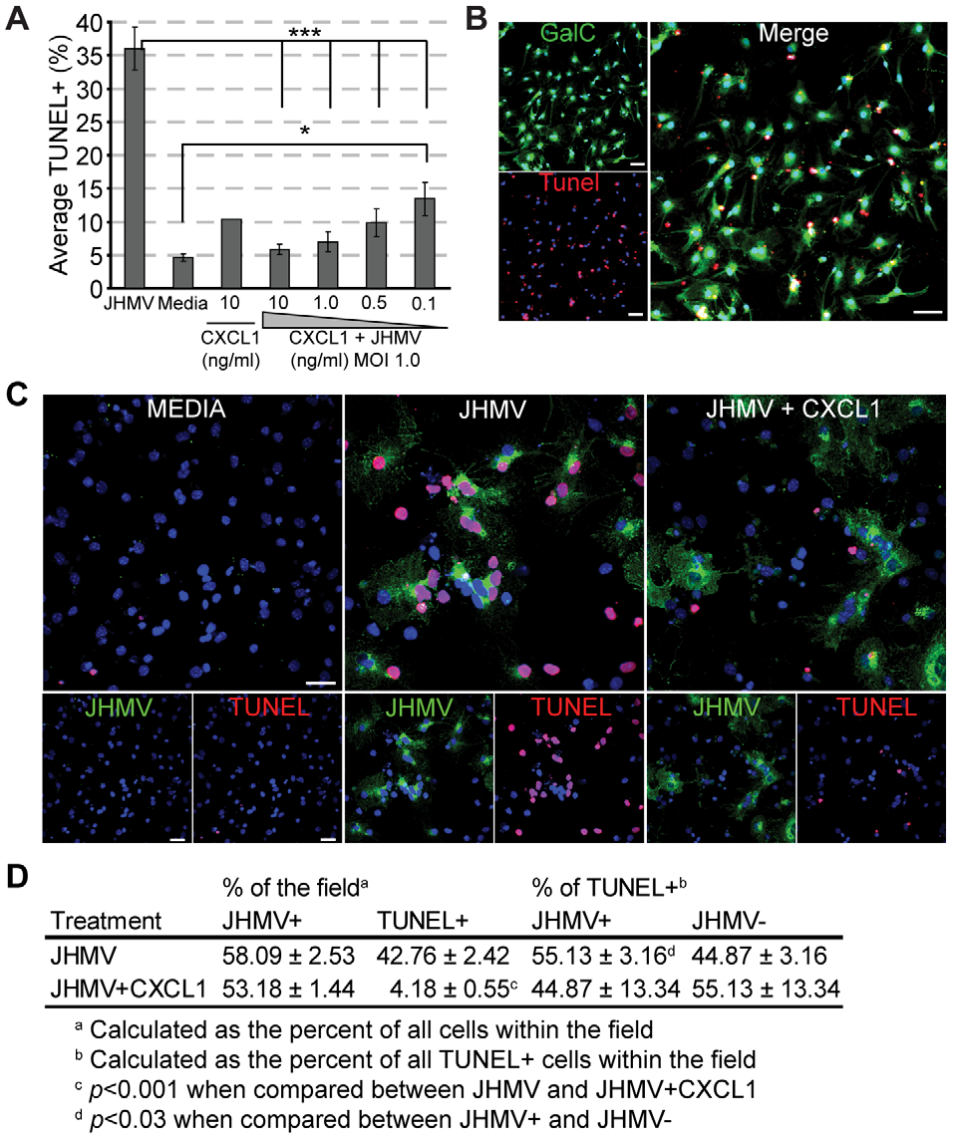
The CXCR2 ligand CXCL1 protects oligodendrocytes from JHMV – mediated apoptosis *in vitro*. Differentiated oligodendrocyte enriched cultures were infected with JHMV (moi 1.0), treated with varying concentrations of recombinant mouse CXCL1, and assessed for apoptosis via TUNEL 24 hours p.i. (**A**) CXCL1 protected oligodendrocytes from JHMV – mediated apoptosis in a dose dependent manner (*** p<0.001, relative to JHMV only; * p<0.05 relative to Media only). (**B**) A representative image of an apoptotic oligodendrocyte following JHMV infection. (**C**) Immuno-staining for JHMV nucleocapsid (green) in addition to TUNEL (red) revealed both apoptotic JHMV infected and uninfected oligodendrocytes; note the absence of apoptosis within JHMV infected oligodendrocytes treated with CXCL1 at 10 ng/ml. (**D**) Quantification of the frequency of JHMV infected cells amongst TUNEL+ cells revealed that slightly greater than half of the apoptotic cells (p≤0.03) were reactive for JHMV nucleocapsid. Data in panels ***A & D*** is a summation of three independent experiments. Data in panels ***B & C*** is representative of three independent experiments. Bars: (**B**) 60 µm; (**C**) 30 µm.

**Figure 8:**
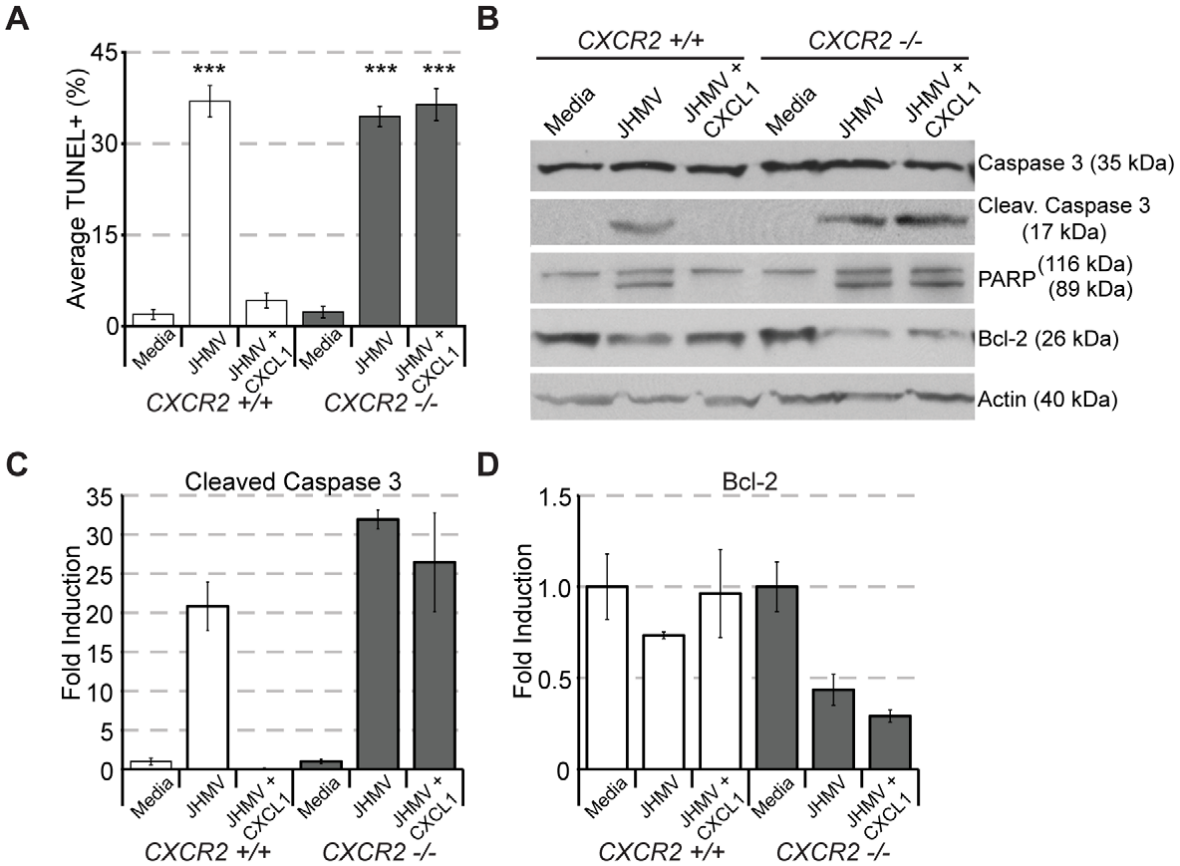
CXCR2 deficient oligodendrocytes are insensitive to CXCL1 – mediated protection from apoptotis. Differentiated *CXCR2+/+* and *CXCR2−/−* oligodendrocyte enriched cultures were infected with JHMV (moi 1.0) and treated with recombinant mouse CXCL1 at 10 ng/ml. (**A**) While CXCL1 protected *CXCR2+/+* oligodendrocytes from JHMV-mediated apoptosis, *CXCR2−/−* oligodendrocytes were not protected (*** *p*≤0.001, relative to Media). Protein lysates were collected from JHMV infected CXCR2 +/+ and *CXCR2−/−* cultures and cleaved caspase 3, the caspase target PARP, and Bcl-2 expression were assessed (**B**). Densitometric quantification of the cleaved caspase 3 and Bcl-2 blots is presented panels **C** and **D**. Data in panels **A–D** is representative of three independent experiments.

Previous reports have indicated that JHMV directly induces apoptosis of infected oligodendrocytes *in vitro* [24]–[27]. Therefore to determine whether CXCR2 signaling can protect against JHMV – mediated apoptosis of oligodendrocytes, an *in vitro* culture system using enriched glial cultures derived from differentiated neural precursor cells (NPCs) was employed. Differentiation resulted in >80% of cells expressing the oligodendrocyte marker GalC; the remaining cells expressed either the astrocyte marker GFAP or neuron maker MAP2 (**Figure S2A**). Differentiated oligodendrocytes also constitutively expressed CXCR2 (**Figure S2B**). Infection of oligodendrocyte – enriched cultures with JHMV (MOI = 1.0) increased TUNEL staining, suggesting that oligodendrocytes were undergoing apoptosis in response to viral infection (**Figure 7A & B**). Indeed, Western blotting confirmed the induction of apoptosis. Following JHMV infection, we observed the cleavage of procaspase 3 (35 kDa) into its active form (17 kDa), in combination with cleavage of the caspase 3 target PARP, and muted expression of Bcl2, a member of the anti-apoptotic Bcl-x family of proteins, confirming previous reports [24]–[27] that *in vitro* JHMV triggers apoptosis within infected oligodendrocyte cultures (**Figure 8B–D**). We next assessed whether or not apoptosis was limited to JHMV infected oligodendrocytes or was more widespread amongst uninfected oligodendrocytes in the culture. Immunostaining for viral nucleocapsid in conjunction with TUNEL revealed that slightly more than half of the TUNEL reactive cells were positive for viral antigen (**Figure 7C & D**). Therefore, although there was a modest but significant (*p*≤0.03) enrichment of viral antigen amongst the apoptotic cells, a large proportion of apoptotic cells were unreactive for viral antigen, suggesting that additional mechanisms for apoptosis exist that are not dependent upon viral replication within the target cell (**Figure 7D**). When recombinant CXCL1 was included within JHMV infected cultures, we observed a significant (*p*≤0.001) dose – dependent reduction in the induction of apoptosis as determined by TUNEL (**Figure 7A**). Moreover, we were unable to detect either the activation of caspase 3 or the cleavage of PARP, while Bcl-2 was restored to control levels, suggesting that CXCL1 is protective with regards to JHMV – induced apoptosis (**Figure 8B–D**). To confirm the importance of CXCR2 – mediated signaling in protection from JHMV – induced apoptosis, oligodendrocyte enriched cultures were derived from *CXCR2^−/−^* mice. JHMV infection of *CXCR2^−/−^* cultures resulted in a similar frequency of TUNEL reactive cells as compared to infected wildtype cells, yet CXCL1 did not protect oligodendrocyte cultures from undergoing apoptosis (**Figure 8A**), and this correlated with the presence of activated caspase 3, cleaved PARP, and no restoration of Bcl-2 expression (**Figure 8B–D**). Collectively these data demonstrate the potent role for CXCL1 and CXCR2 in preventing oligodendrocyte apoptosis *in vitro*.

## Discussion

This study has provided previously unappreciated evidence that CXCR2 signaling protects cells of the oligodendrocyte lineage from apoptosis in mice persistently infected with a demyelinating neurotropic virus. Administration of a blocking antibody specific for CXCR2 resulted in increased clinical disease severity associated with enhanced demyelination. The increase in demyelination was not the result of increased viral load or neuroinflammation, indicating alternative mechanisms were responsible for white matter damage. Indeed, anti-CXCR2 treatment increased the overall numbers of apoptotic cells concentrated within demyelinated white matter. *In vitro* studies confirmed our observations in animal studies and emphasized the importance of CXCR2 signaling in protecting oligodendrocyte – enriched cultures from apoptosis through a mechanism associated with controlling Bcl2 expression. Therefore, these findings highlight the importance of CXCR2 signaling in preferential protection of white matter from demyelination in a model of viral persistence associated with on-going neuroinflammation.

In many diverse instances of CNS infection, inflammation, and injury, including MS, EAE, and Theiler's murine encephalitis virus (TMEV), CXCR2 and/or its ligands are upregulated [10], [11], [17], [28]–[32]. During JHMV infection, the CXCR2 ligands CXCL1 and CXCL2 are significantly upregulated and persist within the spinal cord throughout chronic infection, even in the absence of infectious virus. Additionally, we have observed hypertrophic astrocyte derived CXCL1, consistent with previous observations within actively demyelinating MS and EAE plaques [10], [11], [32].

How CXCR2 signaling participates in initiating neuroinflammatory demyelinating diseases has recently been examined. Using EAE as a model of autoimmune demyelination, Segal and colleagues [28] demonstrated that early expression of CXCR2 ligands is critical in both disease initiation and relapse by promoting the accumulation of CXCR2-expressing neutrophils to the CNS that regulate the permeability of the BBB. Similarly, we have recently shown that during acute JHMV-induced encephalomyelitis CXCR2 ligands are important in host defense by attracting neutrophils into the CNS, facilitating the subsequent loss of BBB integrity that permits the entry of virus-specific T cells [1]. Also, during cuprizone – induced demyelination we [15] reported that neutrophil effector function, mediated through CXCR2, is necessary for the initiation of demyelination. However, unlike these previous reports that demonstrated the CXCR2 signaling axis to be an important determinant of immune cell infiltration or function within the CNS, blocking CXCR2 signaling during chronic JHMV-induced disease did not influence the infiltration of the traditional mediators of JHMV – induced demyelination, namely T – lymphocytes and macrophages [33], [34]. Moreover, although neutrophils comprise a rare population of inflammatory cells, near the limits of detection within the CNS during chronic JHMV infection, their numbers or frequencies were not affected following CXCR2 neutralization.

Our findings reveal that CXCR2 signaling is instead important in limiting the severity of demyelination in JHMV – infected mice. Neutralization of CXCR2 significantly delayed clinical recovery and similarly increased the overall extent of demyelination within the spinal cord, and this was associated with a significant increase in the numbers of apoptotic cells within demyelinated white matter tracts. Apoptotic cells have been previously observed within areas of demyelination during chronic JHMV infection [35], [36], however apoptosis has not been thought to play a major role in pathogenesis [36]. Recent evidence has demonstrated that *in vitro* JHMV induces caspase – dependent apoptosis within oligodendrocytes through Bax translocation into the mitochondria [24], [25], [27]. During chronic JHMV infection, oligodendrocytes represent an important viral reservoir [21], [37], [38]; therefore it is likely that some mechanisms exist *in vivo* to protect oligodendrocytes from JHMV – induced apoptosis. In addition to JHMV, oligodendrocytes are also sensitive to FasL [39], TNFα[40], [41], and IFNγ [42], [43] – induced apoptosis, all of which are detectable during chronic JHMV infection [44], [45]. Oligodendrocyte apoptosis, through FAS and TNFR1 signaling, has been suggested to be important for the induction of demyelination during EAE, as the absence of one or both of these apoptosis signaling receptors upon oligodendrocytes reduces both the severity and onset of clinical symptoms [46]. Furthermore, Gold and colleagues demonstrated that when oligodendrocyte apoptosis is enhanced, both the disability and clinical onset of EAE are exacerbated [47]. Studies in MS postmortem tissue have also indicated that oligodendrocyte apoptosis may be an important pathogenic step in a subset of lesions [48]. During coronavirus infection, apoptosis is not restricted to oligodendrocyte lineage cells; microglia and astrocytes have also been reported to undergo apoptosis within demyelinated lesions [35]. Indeed we also observed microglial and astrocytic apoptosis (**data not shown**), however there is little evidence directly linking either microglial or astrocyte apoptosis to demyelination *in vivo*. Therefore, since mice receiving CXCR2 antiserum experienced enhanced clinical severity and demyelination, we focused upon whether CXCR2 signaling was capable of enhancing oligodendrocyte survival *in vitro*.

To determine whether or not CXCR2 signaling was capable of preventing oligodendrocyte apoptosis, JHMV infected cultures were treated with the CXCR2 ligand CXCL1. JHMV infected cultures readily initiated apoptosis, activated caspase 3 and downregulated Bcl-2. Slightly more than half of all apoptotic oligodendrocytes were reactive for viral nucleocapsid, suggesting additional complex apoptotic mechanisms in addition to JHMV replication, perhaps through glutamate excitotoxicity [49], as viral infections or cytokine stimulation of astrocytes abrogates *in vitro* glutamate uptake [50]–[52]. JHMV can also induce apoptosis without viral replication through spike glycoprotein and FAS interactions [24], [27], therefore JHMV may also be activating apoptosis in the absence of viral replication. Nevertheless, CXCL1 treatment protected oligodendrocyte cultures from JHMV – induced apoptosis, and this was associated with reduced caspase 3 activation and rescue of Bcl-2 expression. Notably, CXCR2 – deficient cultures infected with JHMV were completely insensitive to CXCL1 – mediated protection, indicating that the protection observed following CXCL1 treatment is mediated through CXCR2. Bcl-2, a member of the large BH family of anti – and pro – apoptotic factors, antagonizes Bax/Bak mediated mitochondrial membrane permeabilization, preventing cytochrome C release and the subsequent induction of apoptosis [53], [54]. Within JHMV infected oligodendrocytes, forced overexpression of Bcl-2 or Bcl-xl, a pro – survival Bcl-2 family member, prevented or muted JHMV – mediated apoptosis [25], [26], presumably by interfering with Bax [25], [53], [54]. Additionally, within MS lesions, oligodendrocyte Bcl-2 expression is highly correlative with the presence of remyelination [55].

CXCL1 plays an important role in arresting the migration of oligodendrocyte precursors during development [12], however, whether CXCL1 performs a similar role within the adult white matter remains uncertain. It has been suggested that the CXCL1 associated with MS lesions [10], [11] may prevent oligodendrocyte precursor cell migration into regions of demyelination and thus prevent subsequent remyelination and recovery [56]. We have not observed any aberrant oligodendrocyte trafficking or localization within mice treated with neutralizing CXCR2 antiserum (**data not shown**). In other complementary models of demyelination and recovery the role of CXCR2 remains enigmatic. Our findings are in marked contrast with a recent report that CXCR2 negatively influences remyelination and recovery following autoimmune demyelination. Using a chemical inhibitor of CXCR2, Miller and colleagues [5] observed that mice receiving a CXCR2 antagonist experienced reduced clinical severity and improved remyelination either during EAE or following lysolecithin microinjection-induced demyelination. The authors postulated that the enhanced recovery observed was due to the removal of a migrational blockade and enhanced differentiation of oligodendrocyte precursor cells, however, whether CXCR2 directly inhibited oligodendrocyte remyelination was ambiguous since CXCR2 antagonism also was associated with reduced macrophage/microglia infiltration [5]. During spinal cord development, we found that CXCR2 - deficient mice experienced abnormal myelination as their oligodendrocyte progenitors were unable to properly arrest and spatially localize within the presumptive white matter [12]. Additionally, OPCs in P7 *CXCR2^−/−^* mice exhibited reduced proliferation and lower levels of programmed cell death [12]. Notably, CXCR2 knockout mice (C57Bl/6) also were resistant to cuprizone – mediated demyelination due to, in part, deficient neutrophil effector function [15]. Different from these results, Raine and colleagues [6] reported a protective role for CXCR2 signaling during demyelination. Transgenic overexpression of CXCL1 within astrocytes induced at disease onset during EAE led to a reduction in late stage clinical severity and demyelination, while enhancing remyelination [6]. We observed that during chronic viral induced demyelination, CXCR2 neutralization delays clinical recovery, enhances demyelination, and increases apoptosis within white matter without influencing viral load or neuroinflammation. Our *in vitro* studies demonstrated that CXCR2 specifically protects oligodendrocytes from JHMV – induced apoptosis. In various models of CNS demyelination and inflammation, CXCR2 has been shown to have enigmatic roles during recovery and repair. Therefore, it is plausible that the pro-apoptotic and pro-survival actions of CXCR2 towards oligodendrocytes will prove to be context-dependent. Adding complexity, clarifying the diverse roles of CXCR2 in neuroinflammation will need to incorporate the extent to which neutrophilic inflammation [1], [15], [28] is relevant for the process under study.

Oligodendrocyte loss can represent a feature of MS lesion development. Therefore it is plausible that CXCR2 on oligodendrocytes and astrocytic CXCL1 near MS lesions [10], [11] may function to promote oligodendrocyte survival. Taken together, we propose a potentially novel role for the chemokine receptor CXCR2 during chronic inflammatory conditions: not as a cellular chemoattractant that influences immune cell infiltration, but as a potent protective stimulus, preventing apoptosis.

## Materials and Methods

### Virus and mice

Age-matched 5–6 week old C57BL/6 mice (C57BL/6, H-2^b^, National Cancer Institute, Frederick, MD) were infected intracranially (i.c.) with 500 plaque forming units (PFU) of MHV strain J2.2v-1 (JHMV) in 30 µl of sterile HBSS. Control (sham) animals were injected with 30 µl of sterile saline alone. Mice were sacrificed at various days post - infection (p.i.) and brains/spinal cords removed and processed for analysis. For analysis of viral titers, one-half of each brain or the entire spinal cord was homogenized and used for standard plaque assay on the DBT mouse astocytoma cell line [57] at the indicated days p.i. All experiments were approved by the University of California, Irvine Institutional Animal Care and Use Committee protocol # 1998–2022.

### Antibody production and administration

Rabbit polyclonal antiserum was generated to a 17-amino acid portion of the amino-terminus ligand binding domain of CXCR2 (MGEFKVDKFNIEDFFSG) [58]. The CXCR2 antiserum specifically blocks CXCR2 dependent infiltration of neutrophils into the peritoneum of mice following thioglycollate irritation [59] and does not bind rabbit complement and deplete neutrophils *in vitro* [1], [28]. For *in vivo* neutralization during JHMV infection, 0.5 ml of anti-CXCR2 or control normal rabbit serum (NRS) was administered intraperitoneally (i.p.) on days 12, 14, 16, 18, and 20 p.i.

### Semi-Quantitative Real Time PCR

Total cDNA from the spinal cords of sham and JHMV infected mice at days 1, 3, 7, 12, 15, 18, and 21 p.i. was generated as previously described [45]. Real-time Taqman analysis for HPRT, CXCR2, CXCL1, CXCL2, and CXCL5 was performed using a BioRad (Hercules, Ca) iCycler with previously described primers and probes [60], [61]. CXCR2 and CXCL1, -2, &-5 expression was normalized to HPRT. Probes were purchased from Integrated DNA Technologies (Coralville, IA), and primers were purchased from Invitrogen (Carlsbad, CA). iQ Supermix (BioRad) was used for all reactions. Assay conditions were as follows: a 4.5 min - at 95°C, and 45 cycles of 30 sec at 95°C and 1 min at 58°C. Data were analyzed with BioRad iCycler iQ5 and quantified with the Relative Expression Software Tool [62].

### Ribonuclease Protection Assay

Chemokine mRNA transcripts were analyzed as previously described [33]. Briefly, total RNA isolated from the brains of anti-CXCR2 and NRS treated mice were analyzed with the mCK5c probe set (BD Biosciences) according to manufacturer instructions. For quantification of signal intensity, individual chemokine band intensity was normalized to L32 control using Image J (NIH).

### Cellular Infiltration Analysis

Flow Cytometry was performed as previously described [33], [63], [64]. Isolated cells were Fc blocked with anti-CD16/32 1∶200 (BD Biosciences, CA) and immunophenotyed with fluorescent antibodies (BD Biosciences) specific for the following cell surface markers: CD4 (L3T4), CD8b (53-5.8), CD8a (53-6.7), Ly6G (1A8), CD11b (M1/70), CD45 (30-F11, E Biosciences), and F4/80 (CI:A3-1, Ab Direct, NC. Appropriate isotype antibodies were used for each antibody. For determination of viral specificity, isolated CNS cells were stimulated *ex vivo* for 6 h with 5 µM of the immunodominant CD4 epitope M_133–147_ [65] or the immunodominant CD8 epitope S_510–518_ [66] and GolgiStop (Cytofix/Cytoperm kit, BD Biosciences), and the production of IFNγ was determined by intracellular staining. Cells were Fc blocked with CD16/32 and stained with FITC – or APC – conjugated CD4, CD8a, or CD8b antibodies (BD Biosciences) before being fixed and permeablized with the Cytofix/Cytoperm kit and stained with PE – conjugated IFNγ (XMG1.2, BD Biosciences). Appropriate isotype antibodies were used for each antibody. Cells were run on a FACStar flow cytometer (BD Biosciences) and analyzed with FlowJo software (TreeStar, OR).

### Clinical Severity and Histology

Clinical severity was assessed using a previously described 4 point scoring scale [33]. **S**pinal cords from 4% paraformaldehyde perfused mice were removed and fixed overnight in 4% paraformaldehyde at 4°C. Spinal cords were separated into twelve 1.5 mm coronal sections, cryoprotected in 20% Sucrose and embedded in OCT. Seven micron thick coronal sections were cut along the length of the spinal cord and stained with luxol fast blue. Areas of total white matter and demyelinated white matter were determined with Image J (NIH). Data is expressed as the percent of total demyelination along the entire length of the spinal cord.

### Immunofluorescence and TUNEL

Tissues were fixed and processed as above. For immunofluorescence, slides were fixed in 4% paraformaldehyde and blocked with 10% Normal Donkey Serum and 0.3% Triton-X 100. Primary antibodies were added serially overnight at 4°C: chicken anti – mouse GFAP 1∶1000 (Abcam, MA), goat anti – mouse CXCL1 (2 ug/ml, R&D Systems, MN), rabbit anti – cleaved caspase 3 rabbit 1∶400 (Cell Signaling, MA), mouse anti – mouse APC (CC1) 1∶20 (Calbiochem, CA), rabbit anti – mouse Olig2 1∶500 (Abcam). Appropriate fluorescent conjugated donkey secondary antibodies were used for visualization (Jackson Immuno, PA). For analysis of *in situ* apoptosis a Fluorescein or TMR-red TUNEL kit was used (Roche) according to the manufacturer's instructions. For co-immunofluorescence to identify activated caspase 3 – positive and TUNEL – positive cells, slides were first TUNEL stained according to manufacturer's instructions and subsequently blocked and assayed with the activated caspase 3 antibody as above. Appropriate TRITC secondary antibodies were then used for visualization (Jackson Immuno). Quantification of TUNEL and areas of white matter was assessed with Image J.

### Isolation of primary oligodendrocyte – enriched cultures

Striatum from postnatal day 1 *CXCR2+/+* and *CXCR2−/−* mice were dissected, triturated, and cultured as previously described [67], [68]. Briefly, dissected striata were razor minced and triturated in prewarmed 0.05% trypsin (Invitrogen) for 10 min. Trypsin digestion was halted with 10 ml of 1X anti-trypsin (Invitrogen). Cells were resuspended in DMEM:F12 (Invitrogen) supplemented with 1X B27, Insulin – Transferrin – Selenium – X (Invitrogen), T3, and 20 ng/ml human recombinant EGF (Sigma) and cultured in low adherent flasks for 5 days. Neurospheres were subsequently collected and transferred into MatriGel coated flasks (BD Biosciences). One day after transfer, EGF was omitted from the media to induce differentiation. Two days after EGF starvation, cells were trypsinized and replated onto MatriGel coated 6 well plates at 1e6 cells/well. Cultures were incubated a further 1–2 days before being assayed. To assess culture purity and CXCR2 expression, 4 day differentiated cultures were washed in cold HBSS and fixed in 4% paraformaldehyde and blocked with 10% goat serum. Primary antibodies were added serially overnight: mouse anti – mouse Gal/C mouse 1∶500 (Chemicon, CA), mouse – anti mouse Map2 1∶750 (Sigma, MO), rabbit anti – bovine GFAP rabbit 1∶500 (Invitrogen), or rabbit anti – mouse CXCR2 antiserum 1∶500. Appropriate conjugated goat secondary antibodies were used for visualization (Invitrogen). To assess apoptosis, cultures were similarly washed and fixed in 4% paraformaldehyde before being subjected to a TMR-red TUNEL kit (Roche) according to manufacturer instructions. Viral infected cells were detected within TUNEL stained cultures using a mouse monoclonal antibody anti-JHMV (J.3.3, 1∶20) directed against the carboxyl terminus of the viral nucleocapsid protein overnight at 4°C. Appropriate goat Alexa 488 anti-mouse secondary antibodies (Invitrogen) were applied, and nuclei were stained with DAPI. Sections were analyzed using an Olympus Fluoview (FV1000) laser scanning confocal microscope, and Volocity image analysis software (Improvision) was used to generate and analyze confocal images. The number of Tunel-positive and JHMV-positive cells relative to the total number of cells was determined from three to five randomly selected fields from three independent experiments.

### Immunoblotting

For western analysis differentiated primary oligodendrocyte enriched cultures were infected with JHMV (MOI 1.0) for one hour. Cells were washed and treated with or without recombinant mouse CXCL1 (Peprotech, NJ) for 24 hours. Cultures were lysed in RIPA buffer (50 mM TrisHCl pH 7.4, 175 mM NaCl, 5 mM EDTA, 1% NP-40, 0.1% SDS, 0.5% DOC) supplemented with protease and phosphatase inhibitors (Roche). Protein extracts (50 ug) were separated on 12% SDS/PAGE, transferred to PVDF membranes and blocked at room temperature with PBS 0.05% Tween-20 plus 5% non-fat milk, then incubated with primary antibodies overnight at 4°C: rabbit anti – total caspase 3 (1∶1000), rabbit anti – cleaved caspase 3 (1∶1,000), rabbit anti – PARP (1∶1,000; Cell Signaling, MA), goat anti – Bcl-2 (1∶5,000; R&D System, MN), and mouse monoclonal actin (1∶50,000; Millipore, MA). Immune complexes were detected using appropriate peroxidase-conjugated secondary antibodies (1∶25,000; Jackson Immuno) and then exposed to a chemiluminescent reagent (SuperSignal West-Pico, Pierce). Densitometric analysis was performed within the linear range with Image J (NIH) and normalized to actin levels.Results were normalized to respective control conditions.

### Statistical analysis

All data is presented as average ± SEM. Statistically significant differences were assessed by one way ANOVA, and *p* values less than 0.05 were considered significant.

## Supporting Information

Table S1 Frequency of inflammatory cell accumulation within the CNS is not altered following CXCR2 neutralization.
(0.03 MB DOC)

Figure S1 CXCR2 neutralization does not affect Chemokine mRNA expression. Chemokine mRNA expression within the brains of anti-CXCR2 or NRS treated JHMV and sham infected mice was assessed at days 15 and 21 p.i. via ribonuclease protection assay (A). Each lane indicates an individual mouse. Quantification of band intensities (B) reveals no significant differences in chemokine mRNA expression between anti-CXCR2 and NRS treated mice.
(0.70 MB TIF)

Figure S2 Oligodendrocytes derived from neural precursor cells express CXCR2 *in vitro*. Neural precursor differentiation produced enriched cultures of Gal/C+ oligodendrocytes, compared to GFAP+ astrocytes or MAP2+ neurons (A). Immunostaining of the differentiated oligodendrocytes revealed CXCR2 expression in vitro (B). Data in panel A is a summation of two independent experiments. Representative image is shown in panel B.
(0.89 MB TIF)
