# Supplementary material for: CXCR2 Signaling Protects Oligodendrocytes and Restricts Demyelination in a Mouse Model of Viral-Induced Demyelination
Source: PLoS One. 2010 Jun 28;5(6):e11340. doi: 10.1371/journal.pone.0011340 (PMC2893165; doi:10.1371/journal.pone.0011340)
Supplement: Table S1 — Frequency of inflammatory cell accumulation within the CNS is not altered following CXCR2 neutralization. (0.03 MB DOC) [file pone.0011340.s001.doc]

**Supplementary Table 1. Frequency of inflammatory cell accumulation within the CNS is not altered following CXCR2 neutralization**

|  |  |  | Days Post Infection^b^ | |
| --- | --- | --- | --- | --- |
| Treatment | Infiltrate | Tissue | 15 | 18 |
| anti-CXCR2^a^ | CD4 | Brain | 22.85 ± 1.19^c^ | 18.03 ± 0.84 |
|  | CD8 | Brain | 7.50 ± 0.40 | 8.00 ± 0.75 |
|  | Macrophage | Brain | 6.28 ± 0.58 | 6.38 ± 0.43 |
|  | Neutrophil | Brain | 1.21 ± 0.12 | 1.71 ± 0.34 |
|  | Neutrophil | Spinal Cord | 0.64 ± 0.14 | 0.61 ± 0.12 |
| NRS | CD4 | Brain | 21.65 ± 0.92 | 18.04 ± 1.36 |
|  | CD8 | Brain | 7.39 ± 0.32 | 9.90 ± 1.16 |
|  | Macrophage | Brain | 6.26 ± 0.25 | 6.83 ± 0.55 |
|  | Neutrophil | Brain | 1.24 ± 0.16 | 1.19 ± 0.11 |
|  | Neutrophil | Spinal Cord | 1.00 ± 0.47 | 1.17 ± 0.18 |

^a^ n=5-6 for both treatment groups

^b^ Data is representative of 2-4 independent experiments

^c^ Data is presented as average frequency ± SEM
