# Supplementary figures and images for: CXCR2 Signaling Protects Oligodendrocytes and Restricts Demyelination in a Mouse Model of Viral-Induced Demyelination
Source: PLoS One. 2010 Jun 28;5(6):e11340. doi: 10.1371/journal.pone.0011340 (PMC2893165; doi:10.1371/journal.pone.0011340)

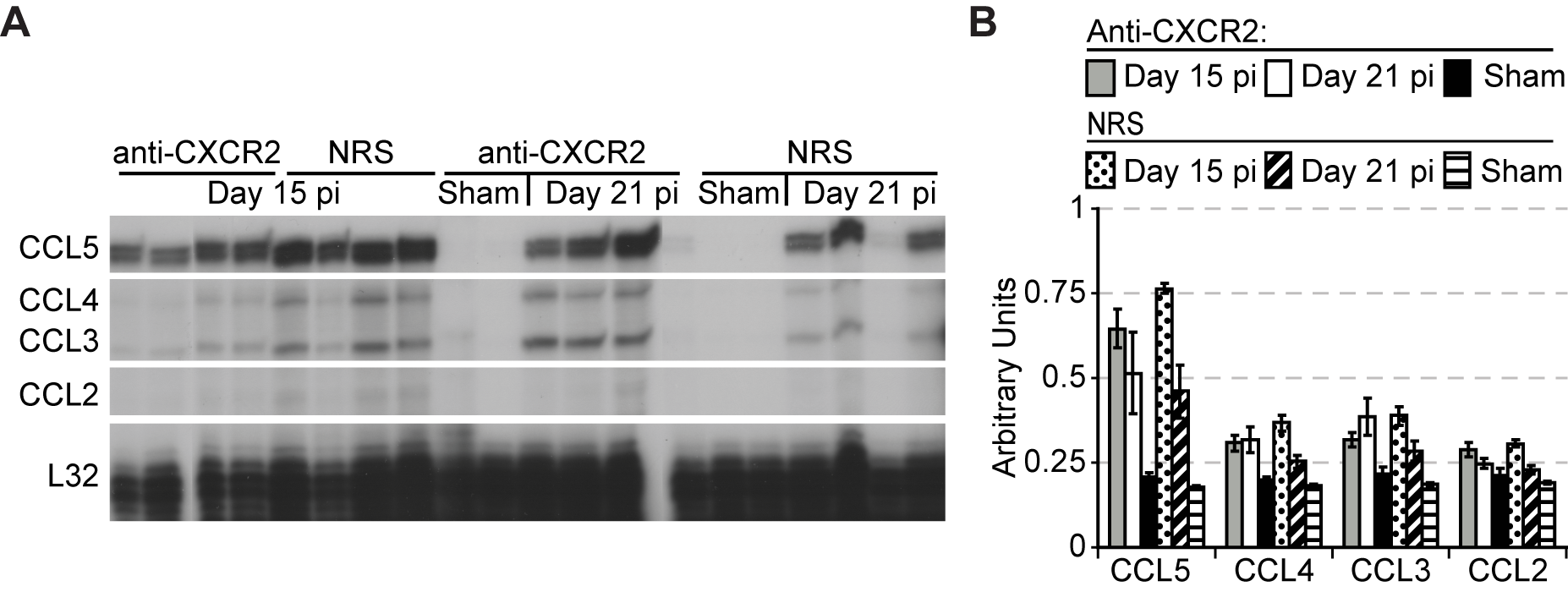

Supplement: Figure S1 — CXCR2 neutralization does not affect Chemokine mRNA expression. Chemokine mRNA expression within the brains of anti-CXCR2 or NRS treated JHMV and sham infected mice was assessed at days 15 and 21 p.i. via ribonuclease protection assay (A). Each lane indicates an individual mouse. Quantification of band intensities (B) reveals no significant differences in chemokine mRNA expression between anti-CXCR2 and NRS treated mice. (0.70 MB TIF) [file pone.0011340.s002.tif]

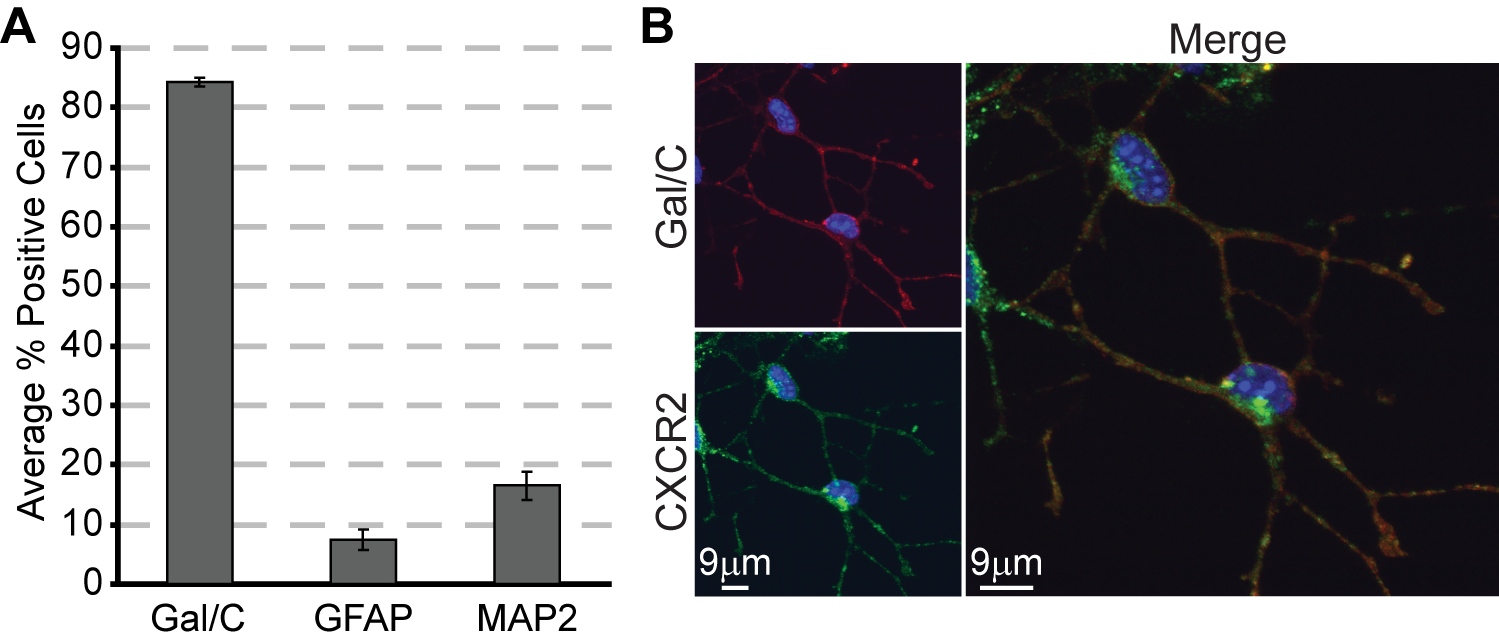

Supplement: Figure S2 — Oligodendrocytes derived from neural precursor cells express CXCR2 in vitro. Neural precursor differentiation produced enriched cultures of Gal/C+ oligodendrocytes, compared to GFAP+ astrocytes or MAP2+ neurons (A). Immunostaining of the differentiated oligodendrocytes revealed CXCR2 expression in vitro (B). Data in panel A is a summation of two independent experiments. Representative image is shown in panel B. (0.89 MB TIF) [file pone.0011340.s003.tif]
